# 

**Published:** 1857-11

**Authors:** 


					﻿No. VI.]
November.
[Vol. XIII.
BUFFALO
MEDICAL JOURNAL
and
IMvntljh) Me view
(IT
MEDICAL AND SURGICAL SCIENCE.
SANFORD B. HUNT, M. I).,
EDITO ZFt _
AUSTIN FLINT, Jr., M. 1).,
ASSOCIATE EDITOR.
BUFFALO, N. ¥.:
E. R. J E W ETT <fc 0 O. ’ S STEAM PRESS.
Commercial Advertiser BnildincR.
1857.
Price $2.00 in advance- or $2.50 at the end of three months.
				

